# Conserved N-Terminal Negative Charges Support Optimally Efficient N-type Inactivation of Kv1 Channels

**DOI:** 10.1371/journal.pone.0062695

**Published:** 2013-04-24

**Authors:** Alison Prince, Paul J. Pfaffinger

**Affiliations:** Department of Neuroscience, Baylor College of Medicine, Houston, Texas, United States of America; Weizmann Institute of Science, Israel

## Abstract

N-type inactivation is produced by the binding of a potassium channel's N-terminus within the open pore, blocking conductance. Previous studies have found that introduction of negative charges into N-terminal inactivation domains disrupts inactivation; however, the Aplysia AKv1 N-type inactivation domain contains two negatively charged residues, E2 and E9. Rather than being unusual, sequence analysis shows that this N-terminal motif is highly conserved among Kv1 sequences across many phyla. Conservation analysis shows some tolerance at position 9 for other charged residues, like D9 and K9, whereas position 2 is highly conserved as E2. To examine the functional importance of these residues, site directed mutagenesis was performed and effects on inactivation were recorded by two electrode voltage clamp in Xenopus oocytes. We find that inclusion of charged residues at positions 2 and 9 prevents interactions with non-polar sites along the inactivation pathway increasing the efficiency of pore block. In addition, E2 appears to have additional specific electrostatic interactions that stabilize the inactivated state likely explaining its high level of conservation. One possible explanation for E2's unique importance, consistent with our data, is that E2 interacts electrostatically with a positive charge on the N-terminal amino group to stabilize the inactivation domain at the block site deep within the pore. Simple electrostatic modeling suggests that due to the non-polar environment in the pore in the blocked state, even a 1 Å larger separation between these charges, produced by the E2D substitution, would be sufficient to explain the 65× reduced affinity of the E2D N-terminus for the pore. Finally, our studies support a multi-step, multi-site N-type inactivation model where the N-terminus interacts deep within the pore in an extended like structure placing the most N-terminal residues 35% of the way across the electric field in the pore blocked state.

## Introduction

N-type inactivation is one of the fundamental gating mechanisms found in voltage-gated potassium channels that acts to shut down channel function during a sustained depolarization [1], [2]. This autoinhibitory phenomenon provides a negative feedback mechanism to self regulate the channel's function, as well as providing a signal that contains information about the recent past history of membrane depolarization in the cell [3], [4]. Extensive biophysical analysis has revealed that during N-type inactivation a portion of the channel's N-terminus enters the activated channel's transmembrane pore, binding within and thus blocking ion conduction [5]. The recovery from N-type inactivation occurs at negative membrane potentials where the unbinding of the peptide allows the channel to close, sterically occluding access of the N-terminus to the pore binding site [6]. Since only channels that are unbound are able to close, the process of recovery takes some time, resulting in a slow tail current with kinetics reflecting the rate limiting step of peptide unbinding from the pore [7].

Previous studies have investigated the physiochemical requirements for residues in the N-terminus of voltage-gated potassium channels that are required to produce effective N-type inactivation [8], [9]. Introduction of a single negative charge into the N-terminus of the Drosophila Shaker ShB channel, mutation L7E, is sufficient to completely block the ability of the N-terminus to inactivate the channel [7]. In other N-type inactivation domains, introduction of negative charges by phosphorylation of the N-terminus is sufficient to disrupt N-type inactivation [10], [11], [12]. These results, combined with other mutational studies have led to the general picture that an effective N-type inactivation ball peptide is generally hydrophobic and positively charged, and that negative charges in the N-terminus are disruptive to N-type inactivation [2].

**Figure 1:**
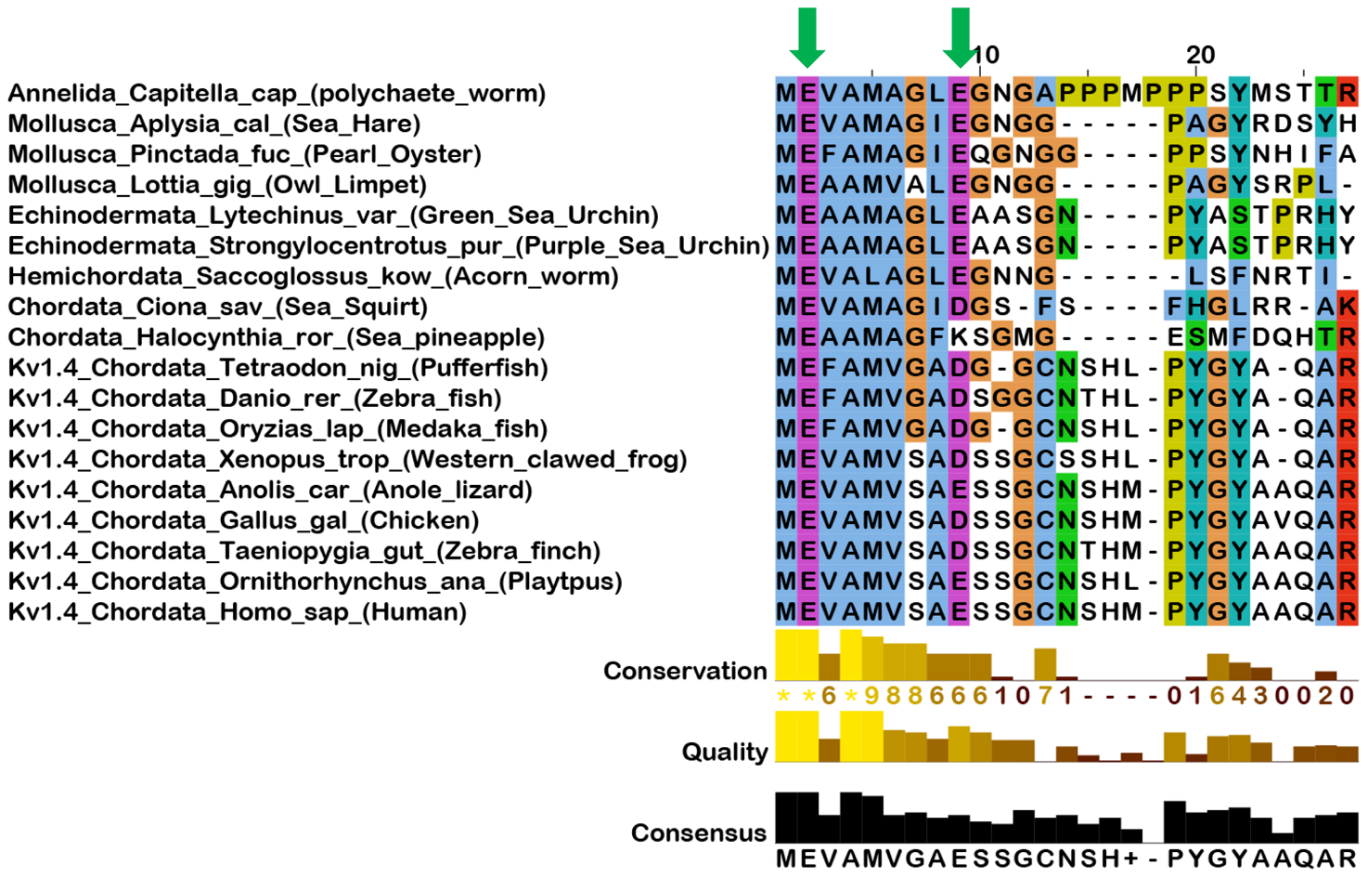
Alignment of Kv1 N-terminal sequences showing the highly conserved negative charges in the N-terminal inactivation domain. Green Arrows show locations of highly conserved negatively charged amino acids at positions 2 and 9. Sequence homology is evident throughout this region of the N-terminus as indicated by the high level of conservation and quality of the alignment. The consensus sequence at the bottom gives an indication of residues that have likely been conserved from the hypothesized Kv1AnID.

It is surprising to note, then, that the human Kv1.4 type channel shows robust N-type inactivation despite the presence of two negatively charged residues in the N-terminus [13]. Furthermore, analysis of other Kv1.4/KCNA4 sequences from a large number of sequenced and assembled vertebrate genomes shows that these same two negatively charged residues are highly conserved (Fig. 1). Indeed, the conservation patterns evident in the N-termini of Kv1.4 channels appear to extend far beyond vertebrates to include sequences from Kv1 channels in annelids, mollusks, hemichordates and urochordates, as can be seen in Fig. 1. While not all of these channels have been biophysically examined for N-type inactivation, the Aplysia Kv1 channel has, and shows N-type inactivation properties remarkably similar to vertebrate Kv1.4 [14], [15]. In addition, the Halocynthia Kv1 has been partially characterized and shows rapid inactivation consistent with N-type inactivation [16]. Although the conservation of negative charges in the N-terminal inactivation domain is strikingly widespread in Kv1 channels, this motif is not found in N-type inactivation domains of other channels, or auxiliary subunits [17] (Figure S1). Based on the unique presence of this sequence motif in Kv1 channels of both protostomes and deuterostomes, it seems likely that it has been inherited in an unbroken chain from an ancestral Kv1 channel N-type inactivation domain, Kv1AnID (**Kv1 An**cestral **I**nactivation **D**omain), that evolved in a very early bilateralian. Indeed, these channels show strong sequence similarity throughout the entire N-terminus prior to the T1 domain (Figure S2). Interestingly, the primitive genes for Kv1 channels containing the Kv1AnID are generally structurally simple, with the amino acid coding sequence often encoded in a single exon, similar to most human Kv1 channels. However, early in the evolution of ectdysosa both the simple gene structure and the Kv1AnID appear to have been lost since most species have complex alternatively spliced Kv1 genes and lack N-termini with these signature negative charges, including the classic Drosophila Shaker channel (Figure S1) [18], [19], [20].

To better understand the roles of the highly conserved negative charges in the Kv1AnID, we have performed a series of mutational studies on the Aplysia Kv1 channel (AKv1) N-type inactivation domain. The AKv1 channel was chosen because it is the most primitive, well studied channel containing a classic Kv1AnID sequence, and does not have the potential complication of a secondary, cryptic inactivation domain found in some Kv1.4 channels [21], [22]. Our results show that these conserved negative charges play a key role in establishing a highly effective and efficient N-type inactivation process that likely underlies their high level of conservation in Kv1 channels throughout the evolution of these very diverse species.

## Materials and Methods

### Ethics Statement

The procedures on animals conducted in this work were performed in strict accordance with Animal Welfare Act, the Public Health Services Animal Welfare Policy, and The National Institute of Health Guide for Care and Use of Laboratory Animals. The experimental protocol was approved by the Institutional Animal Care and Use Committees (IACUC) of Baylor College of Medicine (Protocol Number: AN-752). Following the approved protocol, every effort was made to minimize suffering.

### Molecular Methods

Experiments were performed on the Aplysia Shaker-type voltage-gated potassium channel (AKv1; M95914), which has a well characterized, robust N-type inactivation [14], [23]. The AKv1 coding sequence was cloned into the pBS-II vector (Stratagene). Point mutations were introduced to the AKv1 cDNA using a PCR based method (QuiKChange Kit, Stratagene). The ΔID construct has the entire N-terminal inactivation domain removed by deletion of residues 2–57 using PCR. All mutant constructs were verified by DNA sequencing on both strands through the entire coding sequence. Message RNA was made using the mESSAGEmMACHINE kit (Ambion). Homology analysis was performed on a number of vertebrate and invertebrate Shaker channel N-terminal sequences with a particular focus on domains that have been most extensively analyzed for their N-type inactivation properties. Final alignment was produced using Jalview [24], [25].

### Electrophysiological Recordings

K^+^ currents were recorded from mRNA injected Xenopus oocytes at 1–3 days post injection using a two-electrode voltage clamp (OC-725, Warner Instruments) with currents filtered at 1 kHz (Frequency Devices 902). For most experiments we performed studies in elevated extracellular K^+^ to reduce the effects of C-type inactivation [17]. The normal Hi K bath solution was: (in mM: KCl 98, MgCl2 1, CaCl2 1.8, and HEPES 5 at pH 7.4). DIDS (4,4′-diisothiocyano-2,2′-stilbene disulphonic acid) was added to block endogenous chloride currents. Voltage recording electrodes were pulled on a Sutter Instruments P-97 puller to a resistance of ∼0.5–1 M**Ω** and were backfilled with 3 M KCl. Capacitance and leak currents were removed by off line P/5 leak subtraction. Steady state inactivation and peak activation curves were measured using standard protocols and fit with single Boltzmann curves. Inactivation time course was measured by stepping the oocyte to potentials at least +50 mV or above from a holding potential of −100 mV to strongly activate the outward K^+^ current. Pulses were made long enough to ensure complete N-type inactivation. Recovery from inactivation was measured at potential at least −100 mV or below using a standard two pulse protocol. Typically recovery protocols were performed from an incompletely inactivated state (∼80%) in the first pulse to further limit the development of C-type inactivation during the protocol.

### Data Analysis

Data analysis was performed and graphs generated using a combination of WinWCP, Origin 6.1 (Origin Labs) and Excel (Microsoft) as described previously [17]. Measured time constants are reported as the mean ± SEM (n =  number of independent measurements). Error propagations from measured time constants to energetic estimates were performed using standard propagation formulae [26]. Significance testing was performed using an unpaired two tailed t-tests comparing to wild type. Measured P values are reported, with the significance level set at 0.05.

### Energetic Measurements and Electrostatic Tests

Energetic analyses were performed using a pseudo-1^st^ order model (appropriate for both the MacKinnon and Aldrich models), where ON and Recovery reactions pass through a common rate limiting transition as described previously [17]. Free energy to the transition state for the rate limiting ON and Recovery processes was measured as:
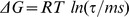
using the appropriate time constant measured in msec, which normalizes our energetics to the fastest relevant process we can measure in our system, 1 kHz. The apparent equilibrium constant for the Free – Bound reaction, K_eq_, is taken as:



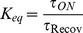
where τ_ON_ is the inactivation time constant (τ_in_) or the adjusted time constant (τ_on_) [17]. Likewise, we estimate the equilibrium energy difference as: 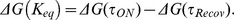


The apparent equilibrium constant for Inactivation at positive potentials (K_I_) was estimated by: 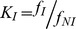



where 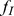 is the fraction of current that is inactivated and 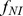 is the fraction not inactivated. Because of the voltage dependence for this equilibrium constant the energetics between different mutants were compared by extrapolation back to 0 mV.

For electrostatic measurements, the free energy of the inactivation ON and Recovery reactions were compared to the charge present at the N-terminal residue being probed (+1 R, K; 0 A, −1 D, E). In addition, double mutations combining charge charges in the channel core and the N-terminus were used to determine the contribution of the core residue site to the electrostatic potential change felt by the N-terminus during the inactivation reaction. A linear relationship between the change in free energy compared to the change in charge is indicative of an electrostatic energy relationship. The slope of the line relating free energy to charge at the test site measures the electrostatic potential change felt by that residue during the transition by: 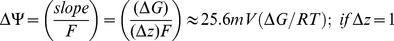



Electrostatic potentials are reported in terms of the potential difference experienced by the N-terminal residue during the examined transition. For electrostatic coupling between an N-terminal residue and a core site, the reported potential is given for a specific charge state of the channel core site being tested compared to neutral residues at this site. The relationship is linear, so the opposite charge at the core site would produce the opposite coupling potential.

### Structural Modeling

Structural modeling was performed using VMD and DeepView on the AKv1 model previously developed from the published Kv1.2 structural model [17], [27], [28], [29]. A surface electrostatic potential model for the AKv1 surface was constructed by mapping the electrostatic potential onto the 1.4 Å probe accessible surface using DeepView.

### Linear Energy Analysis

Linear Energy analysis is based on a generalization of the Brønsted plot from classical physical-chemistry, where mutational effects are transformed into logarithmic energy space and the energetic impact of a mutation on the equilibrium constant for a protein folding reaction is related to the impact on the forward or reverse rate for the reaction by the equation [30]:
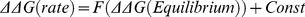



For our studies, mutational effects are studied by comparing the energetic impact on τ_ON_ compared to K_eq_ by:
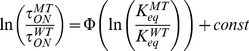



Linear energy analysis assumes that the reaction is progressing incrementally from one distinct end state to another [31], and the mutational effects are modest, such that the reaction still proceeds essentially along the same path with and without the mutation. Under these conditions, the slope factor, Φ, provides important information about the point along the reaction mechanism that the mutation is acting. Plotted points should be bounded by lines with slopes between 0 and 1 that pass through the origin, unless the mutations have a selective effect on an intermediate state that is not seen at the end states. In this case, points might fall outside of the region bounded by the 0 and 1 sloped lines.

## Results

### Conservative removal of negative charges in the AKv1 N-terminus

**Figure 2:**
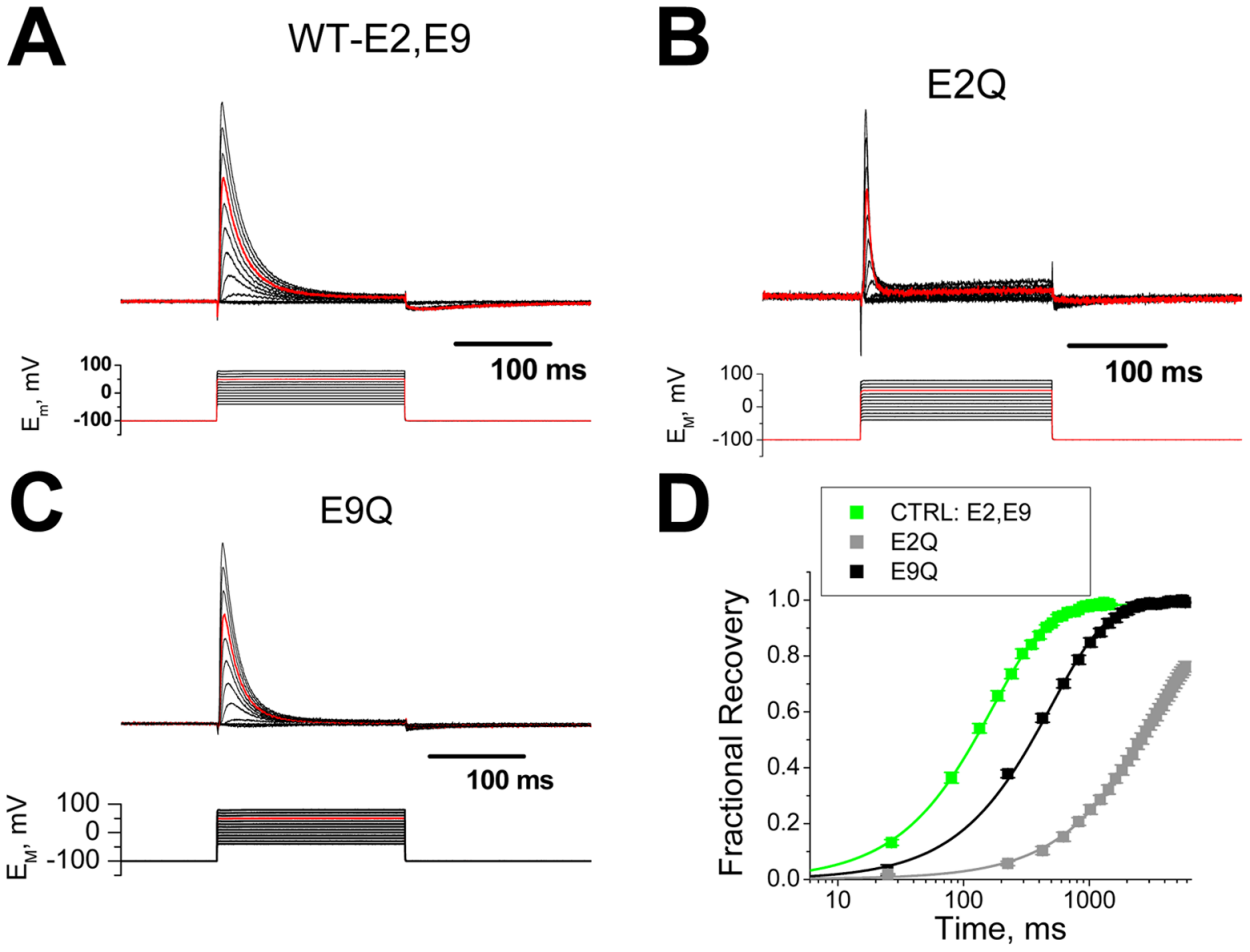
Functional effects of conservative removal of negatively charged residues in the AKv1 N-terminus. Glutamate residues at positions 2 and 9 were mutated to the uncharged but similarly sized glutamine residue. Currents recorded in response to voltage steps from −40 mV to +80 mV, with the step to +50 mV highlighted in red. **A**) Normal kinetics for wild type AKv1 with E2 and E9. Currents show a rapid and almost complete inactivation during strong depolarizations. A small tail current produced by N-terminal unbinding during the recovery process is evident on return of the membrane potential to −100 mV. **B**) The mutation E2Q, removing the negative charge from the second position greatly accelerates the inactivation decay kinetics, but leaves slightly greater levels of sustained current at the end of the pulse. Tail currents during the recovery process at −100 mV are less pronounced than with the wild type channel. **C**) The mutation E9Q, removing the negative charge from the 9^th^ position slightly accelerates inactivation kinetics and slightly increases the level of block at the end of the pulse. Tail currents with this mutation are also less pronounced than wild type. **D**) Analysis of recovery kinetics at −100 mV shows that wild type channels recover most quickly while recovery for the uncharged mutants is dramatically slowed. All recovery kinetics are well fit by a single exponential. (N's: E2, E9(27), E2Q(8), E9Q(5)).

In order to test the functional significance of the conserved negative charges at positions 2 and 9 of the AKv1 Kv1AnID, we introduced the conservative substitution of glutamate (E) to glutamine (Q), which is similar in size but uncharged (Fig. 2). Wild type and mutant channels were expressed in Xenopus oocytes and characterized by two electrode voltage clamp. Fig. 2A shows the characteristic strong and almost complete inactivation seen in the wild type AKv1 channel which contains the conserved E2 and E9 residues. The E2Q mutation produces a channel that inactivates much more rapidly than wild type, although there is slight increase in the level of sustained current (Fig. 2B). The E9Q mutant also appears to inactivate rapidly, although not as fast as E2Q, with very little sustained current at the end of the pulse (Fig. 2C). Analysis of the recovery from inactivation at −100 mV shows that both E2Q and E9Q recover more slowly than wild type (E2, E9: 152±5 ms (n = 27); E2Q: 2285±197 ms (n = 8, P<0.0001); E9Q: 500.7±16.4 ms (n = 5, P<0.0001)). E2Q recovery is more than 10× slower than E2, despite the fact that the residual current at the end of a depolarization is greater (Fig. 2D). This observation contradicts the normal view of recovery from inactivation, since with the larger residual current should indicate that more channels are available to close and thus recovery from inactivation should be faster. We conclude, therefore, that the conservative removal of negative charges from the AKv1 Kv1AnID does not eliminate N-type inactivation, but rather produces channels that inactivate more rapidly and stay inactivated longer during recovery protocols.

### Effect of Varying Residue 2 R-group Size and Charge

**Figure 3:**
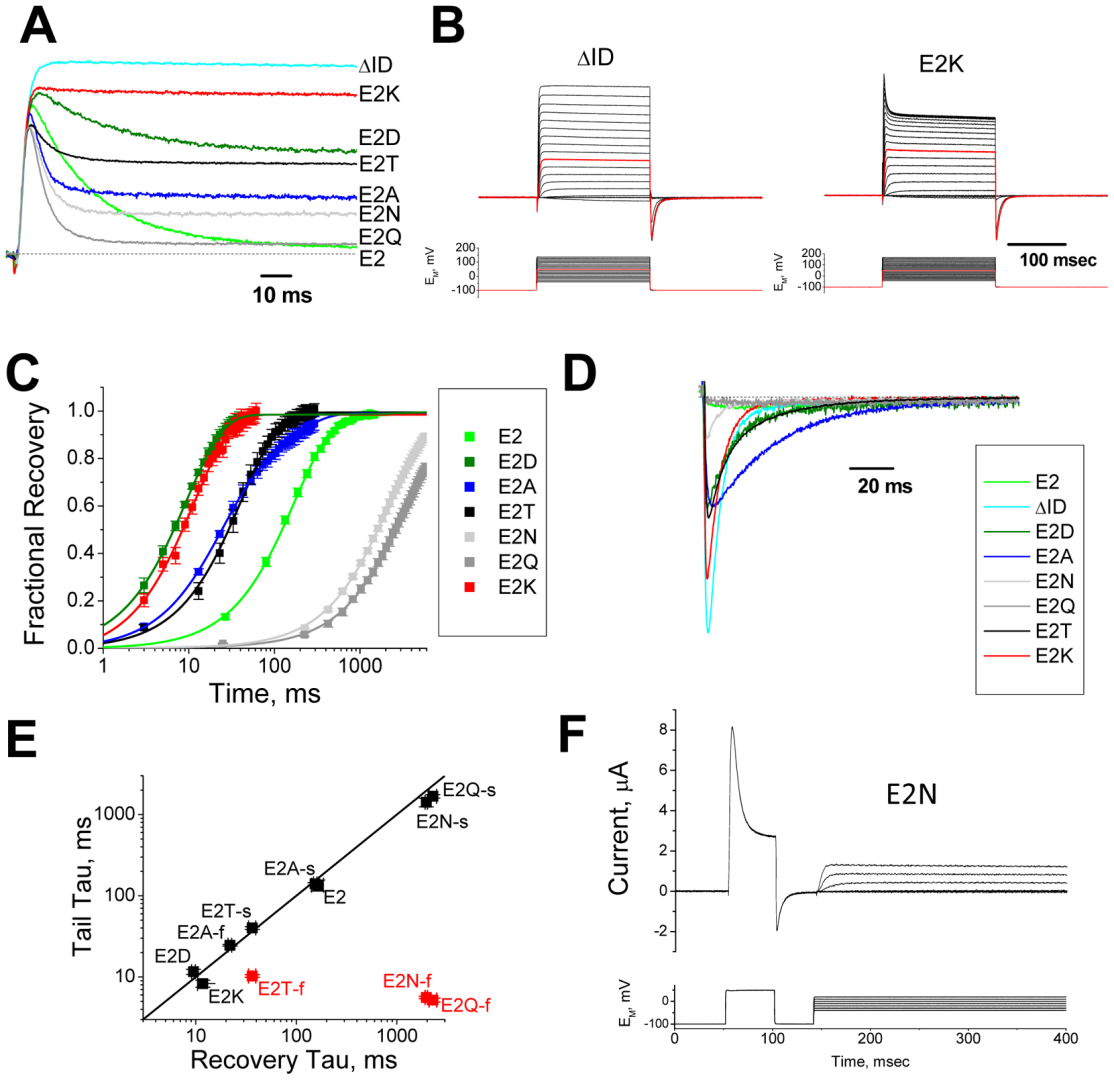
Effects of size and charge changing mutations at position 2 on N-type inactivation. **A**) Typical currents recorded in response to a step depolarization to +50 mV for mutations to residue 2. Current amplitudes were normalized to the same peak rate of rise to compare the sustained currents remaining at the end of the depolarization. ΔID is a construct with the N-terminus removed, eliminating N-type inactivation. **B**) Analysis of inactivation properties for E2K for steps from −40 mV to +170 mV shows that the inactivation kinetic becomes more apparent as the membrane potential is more depolarized. No such effect is seen if the N-terminus is removed (ΔID). **C**) Average rate of recovery at −100 mV is plotted for a series of substitutions at position 2. The rate varies widely depending upon the specific substitution at residue 2, but not on the net charge at this position. Recovery for all constructs is well fit by a single exponential except for E2A where a second, smaller and slower component of recovery is evident. (N's: E2(27), E2D(6), E2N(7), E2Q(8), E2T(10), E2A(11), E2K(8)). **D**) Tail currents for traces shown in (a) are plotted using the same scaling factors. Tail currents become progressively smaller as the recovery kinetics become slower and the amount of block at the end of the pulse becomes larger. For most constructs the sustained current at the end of the pulse closes more slowly than ΔID, indicating a delayed closing produced by continued interactions with the N-terminus; however, for E2N, and to a lesser extent E2Q a normally closing tail current component is seen that appears to be the size expected if these unblocked channels close normally. **E**) Comparison of tail current kinetics with inactivation recovery kinetics at −100 mV. For charged residues E2, E2D and E2K, there is good agreement between the time constant for recovery from inactivation and the single exponential tail current decay kinetics. For uncharged substitutions, a second tail current kinetic component is seen. For E2N, E2Q and E2T, the faster component is not evident in the recovery kinetics, although for E2A both tail current decay components are evident in the recovery kinetics. **F**) Rapid of reopening for E2N in a two pulse protocol reveals a reduced amplitude current that can gate open and closed with no apparent inactivation kinetics. Reopening steps from −40 mV to +20 mV.

Our results suggest that despite the extensive evolutionary conservation seen, negative charges at positions 2 and 9 are not absolutely required for the Kv1AnID to function as an inactivation domain. However, we sought to determine if there are systematic effects of varying R-group size and charge at these positions that might explain their evolutionary conservation patterns. Fig. 3A summarizes the inactivation properties at +50 mV for a series of substitutions at residue 2. In general, the uncharged substitutions E2N, E2A and E2T inactivate more rapidly than wild type (E2) similar to E2Q (E2: 31.3±1.8 ms (n = 12); E2T: 8.0±0.25 ms (n = 8, P<0.0001); E2A: 3.9±0.14 ms (n = 8, P<0.0001); E2N: 4.6±0.25 ms (n = 7, P<0.0001)), but the amount of inactivation changes dramatically depending upon the mutation (Fract Inact: E2: 0.97±0.01 (n = 12); E2N: 0.72±0.03 (n = 7, P<0.0001); E2A: 0.68±0.01 (n = 8, P<0.0001); E2T: 0.32±0.02 (n = 7, P<0.0001); E2Q: 0.90±0.02 (n = 5, P = 0.003)). E2D, which conserves the charge at position 2, inactivates with slower kinetics similar to the wild type E2 residue (E2D: 24.9±1.4 ms (n = 6, P = 0.034)); however, the amount of inactivation produced is much less (Fract Inact: E2D: 0.29±0.06 (n = 3, P<0.0001)). Finally, E2K, which makes the N-terminus more positive, appears to produce hardly any inactivation at all.

To further probe E2K, we produced progressively stronger depolarizations, and found that the more depolarized the potential becomes the more apparent the inactivation produced by E2K becomes (Fig. 3B). This effect becomes most evident at potentials above +50 mV, well beyond the channels activation range which has a midpoint around −15 mV suggesting a voltage-dependent binding of E2K within the channel's transmembrane pore with more positive potentials inside the cell helping to drive E2K into the pore block site. However, the amount of inactivation produced by E2K at the strongest depolarizations tested, up to 150 mV, does not come close to the levels seen with E2 (Fract Inact: E2K (100 mV): 0.08±0.02 (n = 5, P<0.0001). Interestingly the kinetics for inactivation with E2K are faster than E2 despite the fact that the level of inactivation produced is so poor (At 100 mV: E2: 32.0±1.9 ms (n = 12); E2K: 4.2±1.3 ms (n = 5, P<0.0001)). We therefore conclude that the kinetics of inactivation appear to have a consistent relationship to the charge at position 2, but not the level of inactivation that is produced.

Analysis of recovery from inactivation (Fig. 3C) further shows that the charge at position 2 is a poor predictor of the rate of recovery. The substitutions E2D and E2K which have opposite charges are the fastest to recover (−100 mV: E2D: 9.5±0.8 ms (n = 6, P<0.0001); E2K: 12.3±1 ms (n = 8, P<0.0001). Uncharged residues vary over 100× in their rate of recovery. Wild type E2 in fact is in the middle of the substitution series (−100 mV: E2: 152±5 ms (n = 27)) significantly slower to recover than E2A or E2T (−100 mV: E2A(fast component): 24.7±1 ms (n = 11, P<0.0001); E2T: 37.3±1.6 ms (n = 10, P<0.0001)), but much faster than E2N or E2Q (−100 mV: E2N: 1962±53 ms (n = 7, P<0.0001); E2Q: 2285±197 ms (n = 8, P<0.0001)). It is also curious to note that the level of inactivation produced by the different substitutions is an imperfect predictor of the rate of recovery, since constructs such as E2N, which have a relatively large sustained current (Fig. 3A) show a slow recovery from inactivation (Fig. 3C).

To further examine the relationship between recovery and ball peptide unbinding, we examined the tail currents for the different channel constructs at −100 mV (Fig. 3D), where the tails amplitudes are scaled by the same factor as shown in Fig. 3A. Scaled in this manner, the biggest and fastest tail that we see is the non-inactivating mutant ΔID, which lacks an N-type inactivation ball. The other tail currents are smaller and approximately scaled to the size of the residual current seen at the end of the pulse. For charged residues, E2, E2D and E2K, the tail current decay kinetics are all single exponential, and closely approximate the recovery kinetics (Fig. 3E). For uncharged residues, E2A, E2T, E2N and E2Q, there is a double exponential tail, however, the magnitude of the two components vary widely among these mutations (Figure S3). In most cases only the slower component matches the recovery kinetics, the exception being E2A where both kinetic components are apparent in the recovery (Fig. 3E) (E2A(slow recovery component): 229±22 ms (n = 11, P<0.0001)). Although the slow tail component for E2A is only ∼5% of the tail current, when integrated it is predicted to explain 21% of the recovery for this channel, slightly less than the observed 33±1.2%. For E2N and E2Q, the fast kinetic matches the kinetics for ΔID channel closing, suggesting that this fraction of channels is closing normally. However, if we examine the closing and reopening of E2N (Fig. 3F), where these currents are easily followed due to their large amplitude, we see that although the channel appears to close normally, upon reopening the channel appears to be non-inactivating. A similar result is seen for the smaller residual current in E2Q (data not shown). It therefore seems likely that these uncharged residues are not completely abandoning the channel core during the rapid tail current closing phase, but rather are delayed in leaving the vicinity of the pore following channel closure which accounts for the dominance of the slow tail component in the full recovery from inactivation.

### Effect of Varying Residue 9 R-group Size and Charge

**Figure 4:**
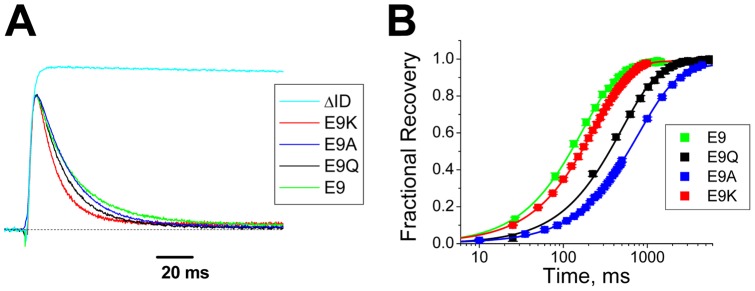
Effects of size and charge changing mutations at position 9 on N-type inactivation. **A**) Typical currents recorded in response to a step depolarization to +50 mV for mutations to residue 9. Current amplitudes are normalized to the same maximum rate of rise as a channel lacking N-type inactivation ΔID. Note the peak amplitudes of these currents are very similar with a consistent acceleration in the decay rate as residue 9 is made more positive. **B**) Average rate of recovery from inactivation at −100 mV is plotted for a series of substitutions at position 9. (N's: E9(27); E9Q(5); E9A(12); E9K(6)). The recovery rate is faster for charged residues at position 9 than for uncharged substitutions.

For substitutions at position 9, the pattern appears to be much simpler. All substitutions tested show strong, almost complete inactivation regardless of charge, however there is a clear progressive change in the inactivation kinetics depending on charge at position 9, which become more rapid as position 9 is made more positive (Fig. 4A) (E9: 31.3±1.8 ms (n = 12); E9A: 17.5±1.2 ms (n = 12, P<0.0001); E9Q: 16.4±1.1 ms (n = 4, P = 0.0004); E9K: 12.1±0.28 ms (n = 5, P<0.0001)). For recovery from inactivation, substitutions at position 9 show a simple correlation with charged residues recovering more rapidly than uncharged residues (Fig. 4B) (E9: 152±5 ms (n = 27); E9A: 701±7 ms (n = 12, P<0.0001); E9Q: 501±16 ms (n = 5, P = 0.0004); E9K: 214±9 ms (n = 6, P<0.0001)). These recovery results suggest that uncharged residues at position 9 are capable of interacting with the channel core in a manner that is not allowed by charged residues. Comparing these observations with the conservation pattern at position 9 (Fig. 1) where E9, D9 and K9 are all accepted substitutions at this position, suggests that evolution is favoring a weaker interaction with the channel core at position 9.

### Linear Energy Analysis for Residue 2 and 9 Substitutions

**Figure 5:**
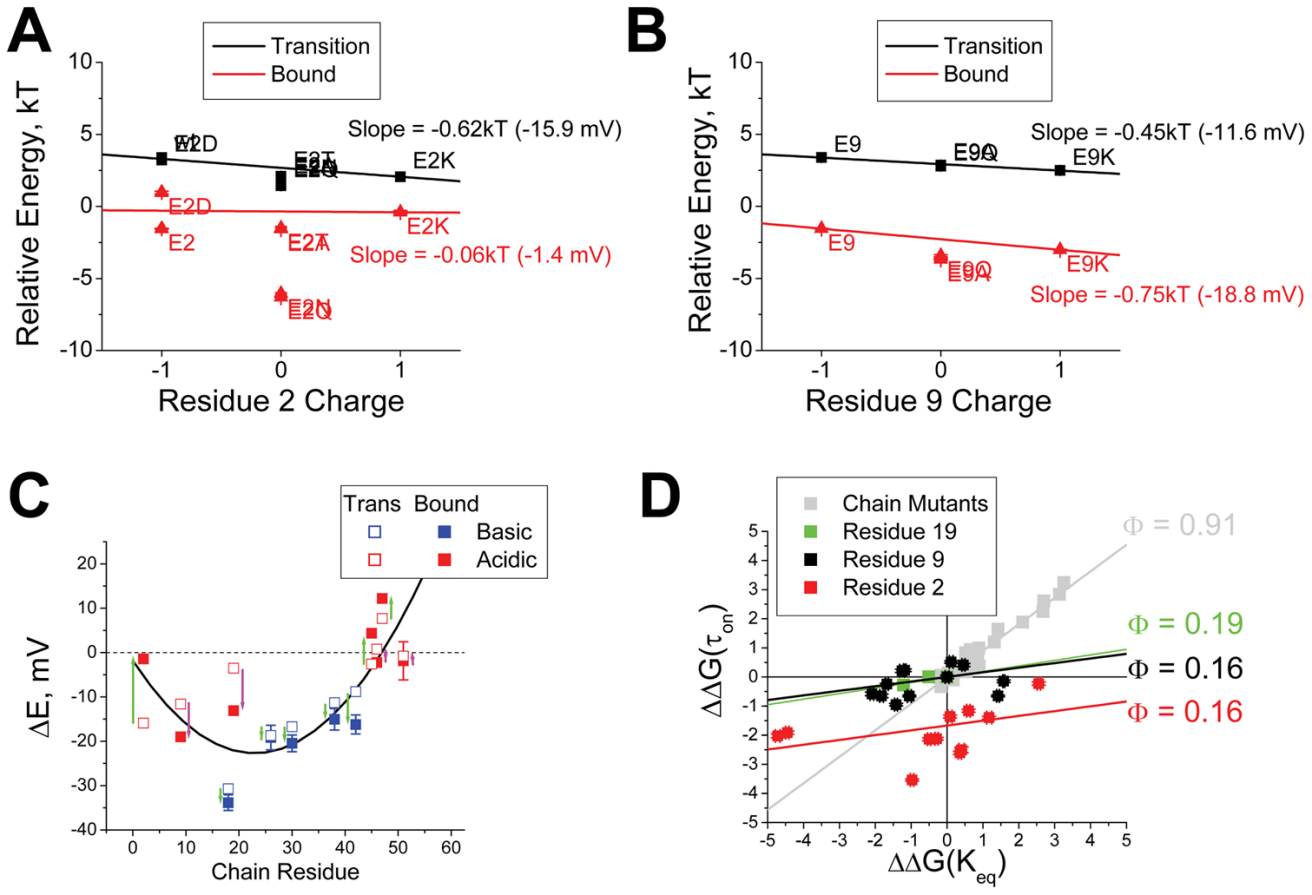
Analysis of Energetic effects of Charge Changing mutations on N-type inactivation. **A**) Relative energy for the rate limiting step to inactivation at +50 mV (Transition) and the difference between this energy and the relative energy for the rate limiting step for recovery from inactivation at −100 mV (Bound) are plotted versus the charge at residue 2. Transition energy lowers as the charge on residue 2 is made more positive, equivalent to a net −15.9 mV electrostatic effect on residue 2 at Transition. There is almost no-consistent electrostatic effect evident when comparing the effects of residue 2 charge on the stability of the Bound state. Uncharged residues show an additional lowering of energy suggesting they are able to make additional favorable interactions in these states that are not available to charged residues. (N's: E2(12), E2D(6), E2N(7), E2Q(6), E2T(7), E2A(12), E2K(10)). **B**) Similar analysis for residue 9 substitutions shows a consistent linear electrostatic effect for residue 9 at Transition that is equivalent to a −11.6 mV electrostatic effect on residue 9. A −18.8 mV electrostatic effect is apparent on residue 9 at the Bound state with additional stability noted for uncharged residues, similar to residue 2 in the transition state. (N's: E9(12); E9Q(4); E9A(5); E9K(6)). **C**) Summary plot of electrostatic effects on residues from 2–55 in the AKv1 N-terminus. Green arrows indicate electrostatic environment changes for residues that are energetically favorable between Transition and Bound, whereas red arrows are changes that are energetically unfavorable. Unfavorable energy changes as inactivation proceeds are seen for most N-terminal negative charges, except for E2, which shows a favorable energetic environmental change between Transition and Bound. **D**) Linear Brønsted plots comparing the energetic impact of mutations on the Transition and Bound states. N-terminal mutational effects, “Chain Mutants,” track the Φ = 1 line, indicating a primary impact prior to Transition, until the most N-terminal negative charges are reached. Then, the curves become much flatter Φ<0.2 indicating the largest impact of mutations at these sites are occurring later in the inactivation cycle near the Bound state. For residue 2 mutations there is an additional vertical shift down from the origin indicative of effects on intermediate steps in the inactivation/recovery cycle.

To determine if there are consistent relationships between inactivation gating properties and the charges of residues at positions 2 and 9, we examined the inactivation reaction energetics for a pseudo-first order reaction cycle (appropriate because N-type inactivation typically has single-exponential inactivation and recovery kinetics), where ON and Recovery reactions pass through a common rate-limiting transition [17]. In this model, the time constant to inactivate provides information about the rate limiting ON Transition step energy while the ratio of the inactivation and recovery time constants provides information about the Bound state energy. In Fig. 5A, we plot these values for residue 2 substitutions versus the charge at position 2. A linear fit to these data shows how the charge at residue 2 changes the value of the inactivation reaction energetics. For inactivation ON, the energy differences between E2, E2D and E2K are consistent with an environment around residue 2 that is −15.9 mV more negative at the Transition state than the Free state. For the Bound state, there is almost no consistent electrostatic effect (−1.4 mV/charge), suggesting that residue 2 has moved beyond the region of negative potential to a more neutral location. Note however, that significant deviations from the fit line are seen for uncharged residues suggesting that they pass through the Transition state more easily than expected and that they have greater affinity for the Bound state than is seen for charged residues.

For residue 9, Fig. 5B, there is a very linear relationship for Transition state energy versus charge suggesting that electrostatic interactions are the primary determinant of mutational differences in the inactivation ON time constant at this position. The data are consistent with an environment around residue 9 that is −11.6 mV more negative at Transition than in the Free state. Our fits to the Bound state data suggest that the environment around residue 9 is even more negative at −18.8 mV, with an additional affinity for non-charged residues. At a first approximation, the environment around residue 9 in the Bound state appears to be very similar to residue 2 in the Transition state, with a similar negative potential and slightly more favorable energy for uncharged residues, possibly indicative of a progressive movement of the chain deeper into the channel between Transition and Bound states.

We can combine this data with results from our previous study on “chain” residues 18–50 by plotting the electrostatic field sensed by the residues at each position in the Transition and Bound states (Fig. 5C) [17]. The arrows indicate the amount of potential change experienced by the residues between the Transition and Bound states. A green arrow indicates that the change is stabilizing for the wild -type residue, while the red arrows indicate the change is destabilizing for the wild-type residue. The general trends for this date are indicated by the parabolic curve fit to the Bound state data set. The results show that there is a point of maximally negative potential experienced by residue 18 that falls off in either direction. For chain residues, the potential changes are almost complete by the time the ON Transition state is reached, with almost all interactions being stabilizing except for the most N-terminal negatively charged residues. For D19, E9 and E2, the potentials the residue experience at the ON Transition become increasingly unfavorable moving towards the N-terminus. In the Bound state, residue D19 and E9 continue to experience progressively more unfavorable potentials, whereas E2 shows a large stabilizing change in environment. This switch from unfavorable environment to more favorable environment for residue E2 may be a key factor that helps drive the strong level of block seen in the normal Kv1AnID's inactivation process.

Fig. 5D shows a summary of all the energetic effects we observed for mutations at these sites and compares these results to our previously analyzed chain mutants in a linear Brønsted plot [17]. The low Φ values for residue 2 and 9 substitutions are indicative that the biggest energetic changes produced by these mutations occur late in the inactivation process, well after the ON Transition. In contrast, the Φ = 0.91 value for chain mutants suggests that the effects of these mutations occur primarily before the ON Transition state is reached. In addition to a low slope, the data appears to indicate a negative y-shift for all residue 2 mutations but E2D. In a general reaction scheme such “catalytic” shifts indicate important effects of these mutations on intermediate states in the pathway.

### Analysis of Electrostatic Coupling Between the Channel Core and Residues 2 and 9

To better understand the electrostatic interactions experience by residue 2 and 9 as well as providing information about the movements of these residues during the inactivation and recovery cycles, we performed a series of mutant cycle experiments between charge changing substitutions in the channel core and charge changing substitutions at positions 2 and 9. Our previous studies on chain residues showed a large contribution of residues EDE161-3 in creating the negative potential experienced by R18. We therefore examined charge changing substitutions there, as well as at position 135, on the top of the T1 domain, which is predicted to be positioned just under the cytoplasmic opening to the transmembrane pore.

**Figure 6:**
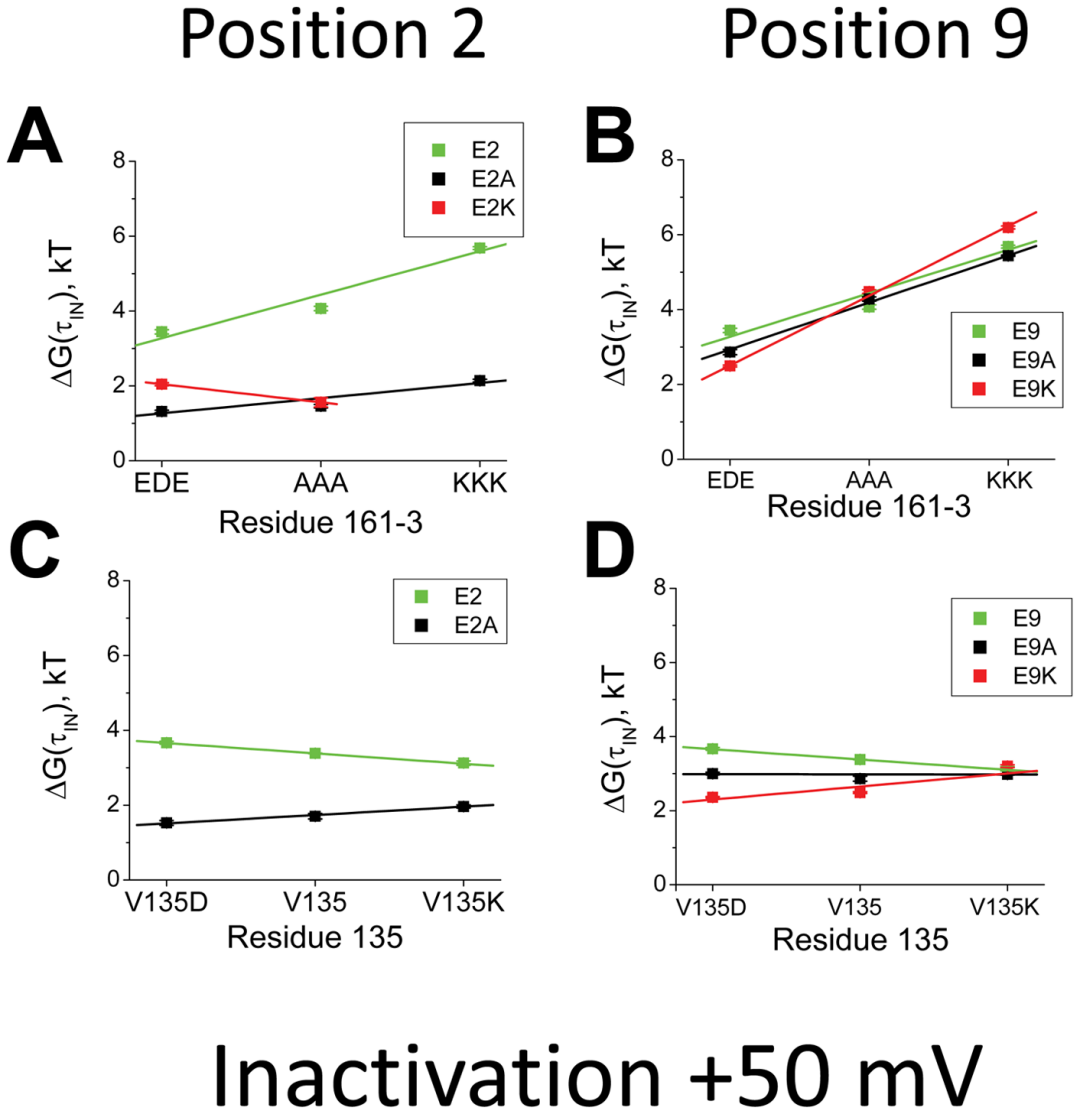
Electrostatic Coupling of residues 2 and 9 with the Channel Core Resides regulates the time constant to inactivate at +50 mV. Energy plots looking at the interactions between charge changing mutations to residues 2 and 9 and charge changing mutations in residues 161-3 and 135 on the time constant to inactivate. **A**) As the charge on position 2 becomes more positive, the effect of mutating 161–3 to more positively charged residues becomes more favorable. This suggests that a stronger interaction between residue 2 and 161–3 slows the rate limiting step in the ON inactivation pathway. (N's: 161-3EDE: E2(17), E2A(8), E2K(5); 161-3AAA: E2(9), E2A(5), E2K(4); 161-3KKK: E2(16), E2A(7)). **B**) If residue 9 is made more positive, the effect of mutating 161–3 to more positively charged residues has an increasingly larger energetic cost for the time constant to inactivate, opposite to the effects seen at position 2. (N's: 161-3EDE: E9(17), E9A(12), E9K(5); 161-3AAA: E9(9), E9A(5), E9K(6); 161-3KKK: E9(16), E9A(8), E9K(5)). **C**) If instead we examine residue 135, at the top of the T1 domain just below the transmembrane pore, then making position 2 more positive increases the energetic cost of placing more positive charges at position 135. (N's: V135D: E2(3), E2A(3); V135: E2(17), E2A(5); V135K: E2(12), E2A(7)). **D**) Adding positive charge at 135 shows an increasingly larger energetic cost when residue 9 is also made more positive. (N's: V135D: E9(3), E9A(4), E9K(8); V135: E9(17), E9A(12), E9K(5); V135K: E9(12), E9A(4), E9K(3)).

Fig. 6A shows the influence of charge changing mutations at residues 161–3 and residue 2 on the energetics responsible for determining the time constant to inactivate. With E2, the rate limiting energy barrier increases as the charge at 161–3 becomes more positive. This effect is due to a combination of effects produced by electrostatic coupling to all the charges on the N-terminus. To isolate the effect produced by coupling specifically to residue 2 we can compare the results obtained with E2 to those obtained with the charge neutralizing mutation E2A. In this case, it is apparent that the slope is more shallow, suggesting that the charge at residue 2 is having a significant impact on the energetic effects of the 161–3 charge changing mutations. Because of the weak inactivation produced by the E2K mutation, this substitution was only examined for a subset of experiments, however, the slope seen with E2K is clearly even more affected in the negative direction further supporting the important role for residue 2 in determining the impact of the 161–3 mutation. In Fig. 6B, we repeat the same experiment only now examining residue 9. Once again, the impact of the 161–3 mutations depends strongly upon the charge being placed on residue 9. However, in this case the slope becomes more positive as residue 9 becomes more positive, similar to what we saw previously with chain mutants, and the opposite of what is occurring with residue 2.

The results for analysis of electrostatic coupling with residue 135 are quite interesting, because for residue 2 the slope becomes more positive as residue 2 becomes more positive, opposite of what we found in the coupling to 161–3 (Fig. 6C). A similar result is obtained for residue 9 and 135 (Fig. 6D), suggesting that the reverse coupling seen in the electrostatic interaction between residue 2 and 161–3 is unique. A simple hypothesis to explain these results is that the energy controlling the time constant to inactivate is strongly influenced by a net translation of residue 2 away from residues 161–3 and towards residue 135 as the ON Transition state is reached while residue 9 is net translating towards both 161–3 and 135.

**Figure 7:**
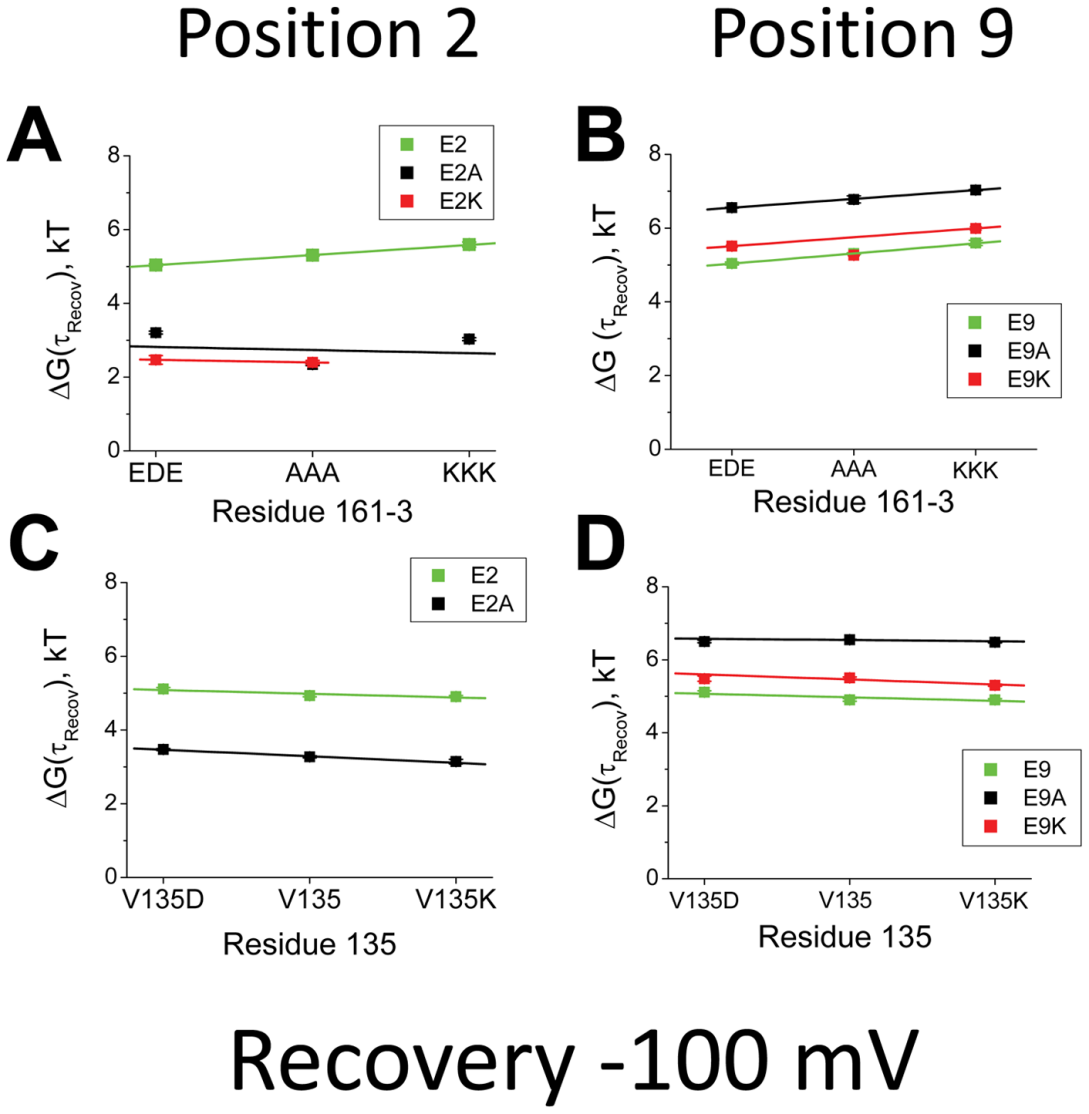
Electrostatic Coupling of residues 2 and 9 with the Channel Core Resides regulates the time constant to recover from inactivation at −100 mV. Energy plots looking at the interactions between charge changing mutations to residues 2 and 9 and charge changing mutations in residues 161–3 and 135 on the time constant to recover from inactivation. **A**) The energetic effects of charge changing mutations at 161–3 on the recovery from inactivation are small and show little evidence for a significant interaction with the charge placed at position 2. (N's: 161-3EDE: E2(27), E2A(7), E2K(8) ; 161-3AAA: E2(6), E2A(6), E2K(4); 161-3KKK: E2(10), E2A(7)). **B**) Similar results are seen with residue 9, where there is little impact of the charge at residue 9 on the small slowing of recovery seen when residues 161–3 are made more positive. (N's: 161-3EDE: E9(27), E9A(12), E9K(6); 161-3AAA: E9(6), E9A(5), E9K(7); 161-3KKK: E9(10), E9A(7), E9K(6)). **C**) Recovery from inactivation also shows little sensitivity to the charge placed at 135, with little impact of the charge at position 2 on this sensitivity. (N's: V135D: E2(3), E2A(3); V135: E2(27), E2A(5); V135K: E2(12), E2A(7)). **D**) Charge on residue 9 shows little effect on the relative impact of charge at position 135. (N's: V135D: E9(3), E9A(3), E9K(4); V135: E9(27), E9A(12), E9K(5); V135K: E9(12), E9A(4), E9K(4)).

**Figure 8:**
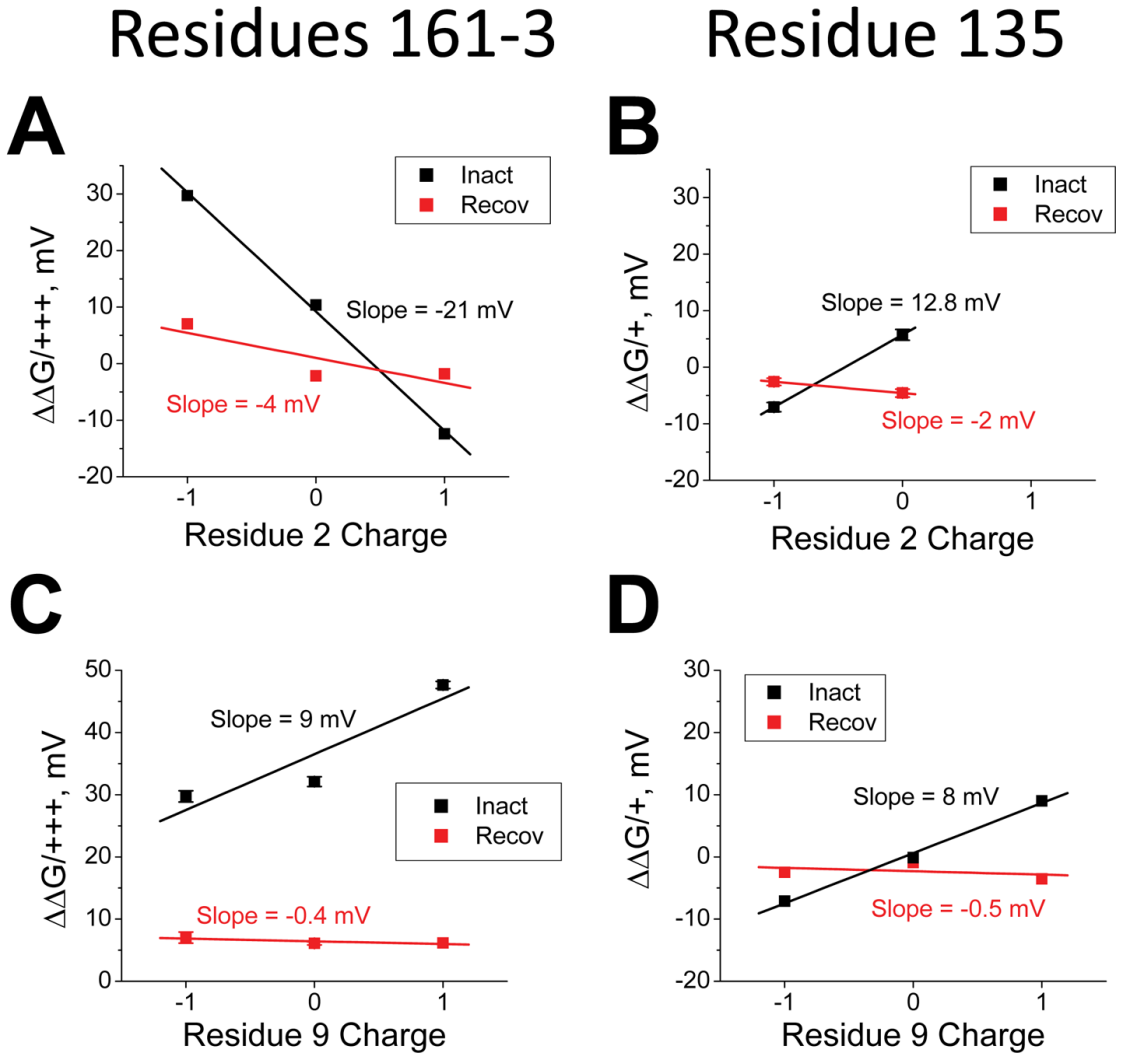
Summary Plots of Electrostatic Coupling of Residues 2 and 9 with the Channel Core Resides 161-3 and 135. Slopes of linear fits to curves in Figs. 6, 7 are plotted versus the charge at positions 2 and 9. **A**) Residue 2 shows a large counterintuitive effect where making 161–3 more positive changes the potential experienced by residue 2 during the rate limiting ON transition by −21 mV. This suggests that residue 2 is net moving away from 161–3 to reach Threshold. Recovery shows only a small coupling of −4 mV suggesting that more positive charges at 161–3 stabilize a negative charge at position 2 in the pore Binding site by 4 mV relative to an uncharged residue. **B**) Residue 2 experiences a 12.8 mV more positive potential during the rate limiting transition as residue 135 is made more positive. There is a small −2 mV coupling of 135 charge to residue 2 in the pore binding site, suggesting a small stabilization is residue 2 is negatively charged. **C**) For residue 9, more positive charges at residue 161–3 increase the electrostatic potential experienced by residue 2 at the Transition state by 9 mV. There is little impact of changing the charge at 161–3 on recovery that is transmitted through the charge at position 9. **D**) More positive charge at 135 similarly increases the electrostatic potential experienced by residue 2 at the Transition state by 8 mV. There is little impact of changing the charge at 135 on recovery that is transmitted through the charge at position 9.

We next examined the electrostatic coupling between residue 2 and 161-3 or 135 during the Recovery process (Fig. 7A–D). Our results show that there is little evidence for significant electrostatic coupling between residue 2 and 161-3 (Fig. 7A) or 135 (Fig. 7C) affecting the energy controlling the kinetics of the Recovery process. The slopes in all cases are almost flat and show little variation with changes in charge at residue 2. For residue 9, we also see little evidence for electrostatic coupling to either 161–3 (Fig. 7B) or 135 (Fig. 7D) affecting the energetics controlling the kinetics of the Recovery process. Summary plots confirm this impression (Fig. 8A–D). For recovery, residue 2 shows weak electrostatic coupling to either 161–3 (−4 mV, Fig. 8A) or 135 (−2 mV, Fig. 8B). Likewise, residue 9 shows almost no coupling to either 161-3 (−0.4 mV, Fig. 8C) or 135 (−0.5 mV, Fig. 8D). While these results might seem somewhat surprising they are in complete agreement with our previous studies on the charged chain residues, where analysis of electrostatic coupling to 161–3 or 135 during the Recovery process also produced flat plots with little variation in slope for all sites tested [17]. The simplest hypothesis that brings all these results together is to propose that reaching the Transition state for Recovery from the Bound state involves little translational movement of the chain, but instead is primarily controlled by the energetics for unbinding within the pore which is far away from either residues 161–3 or 135. Electrostatic coupling to residues 161–3 or 135 likely affects later, non-rate limiting steps during the recovery process and thus the time constant to recover is not affected.

### Electrostatic Interactions of Residues 2 and 9 with the Applied Electric Field

**Figure 9:**
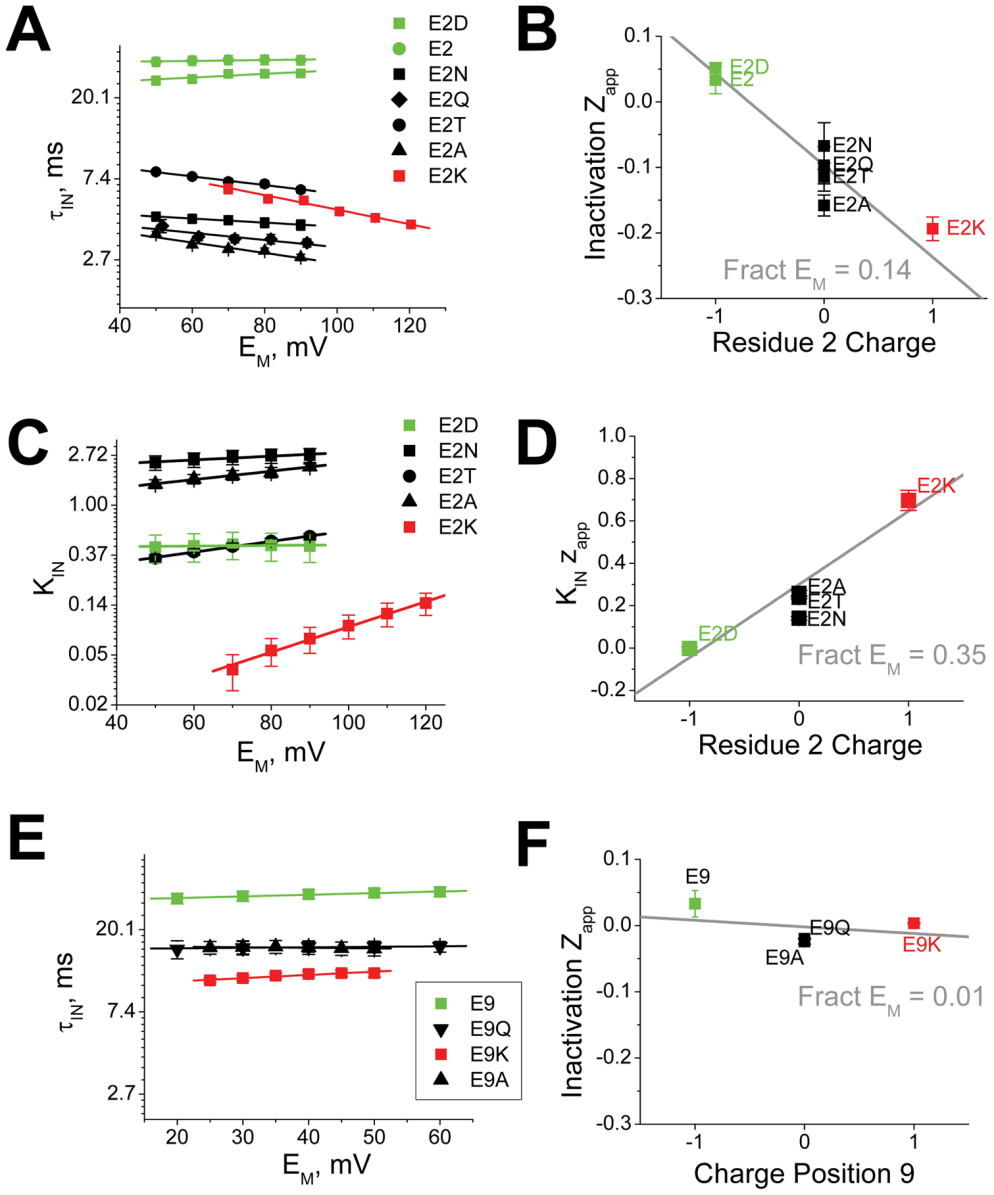
Electrostatic effects of the applied membrane potential on charges present on residues 2 and 9. **A**) Voltage dependence of the time constant to inactivate for different residue 2 mutants at strong depolarizations. As residue 2 becomes more positive, inactivation becomes progressively faster as the membrane becomes more depolarized. (N's: E2(12), E2D(6), E2N(7), E2Q(5), E2T(4), E2A(8), E2K(10)). **B**) Summary plot of the apparent charge dependence for inactivation time constant voltage dependence at residue 2 suggests Threshold occurs when residue 2 is at a position within the pore that experiences 14% of the applied electric field. **C**) Voltage dependence for the blocking affinity of the N-terminus in the pore at large depolarizations. As the charge at position 2 is made more positive the apparent affinity of the N-terminus becomes increasingly better as the membrane potential becomes more depolarized. (N's: E2D(3), E2N(7), E2T(7), E2A(8), E2K(5)) **D**) Summary plot of charge at position 2 versus the voltage dependence of the N-terminus affinity for the pore suggests that residue 2 experiences 35% of the applied electric field at the pore block site. **E**) Charge at residue 9 shows little impact on the voltage dependence for the time constant to inactivate as measured at strong depolarizations. (N's: E9(12); E9Q(4); E9A(5); E9K(6)). **F**) Summary plots show no significant coupling of membrane potential with the charge at position 9, would be expected if residue 9 is outside the transmembrane pore at the Transition state.

The electrostatic effects described thus far are primarily due to the placement of static charges along the inactivation and recovery pathways; however, as shown in Fig. 3B, the applied electric field can also produce electrostatic effects that regulate N-type inactivation. Previous studies suggest that the applied electric field has little electrostatic influence outside the transmembrane pore, but inactivation binding affinity and kinetics can be influenced by N-terminal charges that are located within the pore during the Bound and Transition states. Because inactivation gating is tightly coupled to voltage dependent conformational changes in the channel, we focused on examining voltage-dependent effects at very strong depolarizations where the channel is already fully activated. Fig. 9A shows the voltage dependent effects on the time constant to inactivation at strong depolarizations, well above the −15 mV midpoint of activation. For E2 and E2D the time constant becomes slightly slower as the membrane potential is made more depolarized; however, for uncharged substitutions at position 2, or E2K, the inactivation time constant becomes faster as the membrane potential is made more depolarized. On a semi-log plot, the slope of the line through these points gives the apparent charge (z_app_) that the membrane potential is influencing to produce the observed change in the inactivation time constant. By plotting these z_app_ values versus the charge at residue 2, we can determine the fractional influence of the membrane potential that is transmitted through residue 2 to influence the inactivation time constant (Fig. 9B). The slope of the line indicates that residue 2 is about 14% within the transmembrane electric field at the inactivation ON Transition state.

To compare this to the location of residue 2 within the transmembrane electric field in the Bound state, we looked at whether membrane potential affects the amount of block produced at strong depolarizations. For this analysis we focused on constructs with significant residual currents, because they are less sensitive to small errors in measurement. We then measured the equilibrium constant for conductance block at different potentials (Fig. 9C). It is apparent that the steep voltage dependence for block seen with E2K (Fig. 3B) is reduced for uncharged substitutions, and essentially gone for E2D. We then plotted the z_app_ for these effects versus the charge on residue 2 to determine the fractional influence of the membrane potential that is transmitted through residue 2 to influence the binding of the inactivation domain to the pore (Fig. 9D). The slope of the line to this data suggests that residue 2 is about 35% within the transmembrane electric field when the inactivation domain is in the pore block position within the transmembrane pore. Interestingly, the z_app_ value for both the time constant to inactivate and the inactivation equilibrium constant are near 0 when negatively charged residues are located at position 2. We hypothesize that this occurs because a negative charge on residue 2 neutralizes a positive charge that is already present on the N-terminus in the vicinity of residue 2. The most likely way for this to occur is if there is a positively charged free amino group at the N-terminus of the chain, since the first positively charged side chain does not occur until R18, well outside the pore binding region. Finally, we examined the coupling of membrane potential to substitutions at position 9. Fig. 9E shows the voltage-dependence for the time constant to inactivate depending upon charge at position 9. All substitutions at position 9 show similarly flat traces with little evidence for differences in slope depending upon the charge at position 9. A summary plot (Fig. 9F) shows little systematic change in the z_app_ value showing that almost no energy in the applied membrane potential is transmitted through residue 9 to influence the inactivation time constant, and thus it is likely outside the transmembrane pore at the inactivation ON Transition state.

### Analysis of the Efficiency of N-type Inactivation

**Figure 10:**
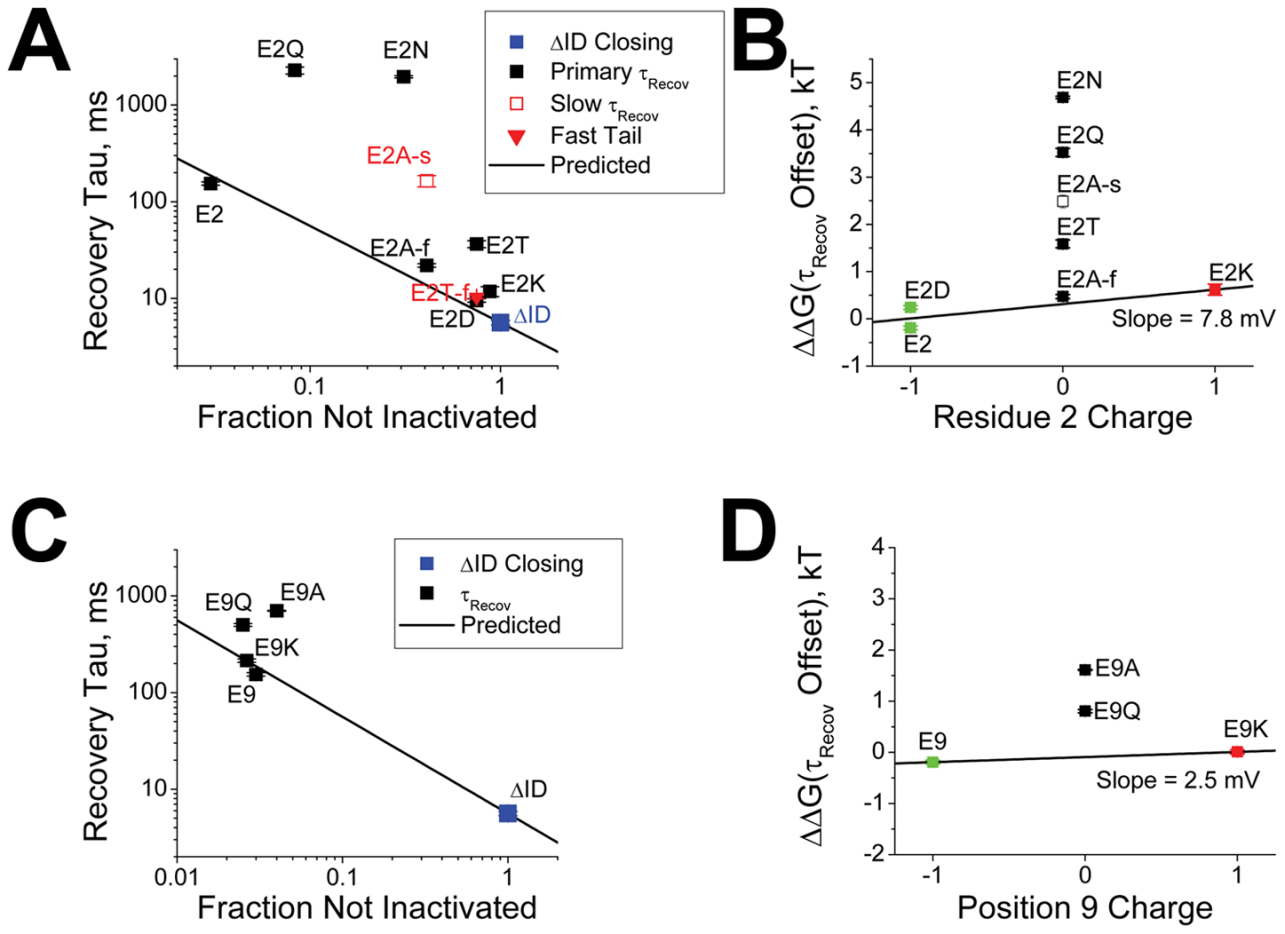
Dependence of Efficiency of Pore block on charges present on residues 2 and 9. **A**) Based on the time constant for channel closing at −100 mV in the absence of N-type inactivation (ΔID), recovery should be slowed by a predictable amount based on the fraction of channels that are unblocked by the N-type inactivation domain (Line:
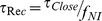
). The wild type residue E2 falls on this predicted line, however, most substitutions lie significantly above the line indicating additional interactions slowing recovery that do not produce channel pore block. (N's: E2(27); E2D(6); E2N(7); E2Q(8); E2T(10); E2A(11); E2K(8)). **B**) The amount of inefficiency in the pore block process produced by different residue 2 mutants can be estimated by measuring how energetically far recovery is from the value expected if recovery were only dependent on the amount of pore block. The charged residues, and faster kinetic components in uncharged residues, fall near the high efficiency 0 kT value. There is a slight systematic effect depending upon charge at position 2, equivalent to 7.8 mV. The slower components of recovery for uncharged residues have large energetic binding components that are slowing recovery without producing additional pore block. **C**) Efficiency analysis for residue 9 substitutions shows again that charged residues are highly efficient at producing pore block with the expected recovery, whereas uncharged substitutions are again shifted above the line. (N's: E9(27); E9Q(5); E9A(5); E9K(6)). **D**) Summary plots show that the charged residues fall near the high efficiency 0 kT value with a slight systematic shift depending upon charge at position 9 of 2.5 mV. Uncharged residues again have interactions with the channel core that are not efficiency being converted into increased pore block.

Classically, slow Recovery from N-type inactivation is thought to be due to the fact that channels that are pore blocked are unable to close. In this model, the time course for recovery from inactivation is primarily determined by the normal rate of channel closing multiplied by the fraction of channels that are able to close. By measuring the fraction of current that is not rapidly inactivating by the N-type mechanism 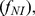 we can readily estimate the predicted recovery time constant 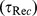 from the time constant to close 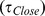 for a channel lacking a blocking N-terminus by: 
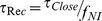
. In Fig. 10A, we compare this predicted curve to the measured time constant to recover for mutations to residue 2. Interestingly, the wild type channel E2 is almost precisely on the predicted curve, whereas most mutants are well above the curve. In Fig. 10B we plot the energetic distance from the predicted curve versus the charge of the residue at position 2. In this curve, despite the large difference in recovery kinetics, both E2 and E2D are close to each other near zero offset. E2K is also near the zero offset line, however it is shifted slightly in the positive direction. This small difference, equivalent to 7.8 mV/charge could be due to a negative electrostatic environment in the pore Block site. Alternatively electrostatic coupling of residue 2 in the pore block site might occur with the S4 domain, similar to a surface charge effect, making the applied membrane potential appear less negative when a positive charge is at position 2. For uncharged residues, two points fall exactly on this line, the fast recovery time constant for E2A and the fast tail current component for E2T, which is not evident in the recovery. All other values show a significant stabilizing offset energy above the expected curve. If pore block is the desired outcome for N-type inactivation, then these offset energies represent inefficiencies in the optimization of the inactivation process.

A similar result is obtained when examining the mutations made to residue 9. Both E9 and E9K are near the predicted recovery tau line (Fig. 10C), but the uncharged mutants are shifted above the line. Plotting the offset energy versus charge at position 9 (Fig. 10D) shows a small shift between E9 and E9K equivalent to only about 2.5 mV/charge of additional stabilization for E9K (again potentially due to a surface charge type effect with S4), with both uncharged mutants showing significant additional stabilization over what is predicted by this theory. Again, if pore block is the desired result of N-type inactivation then these positive offset energies suggest that uncharged mutations at position 9 are less efficient.

## Discussion

Classical experiments on N-type inactivation have suggested that an optimal ball peptide is a hydrophobic structure with closely associated positive charges that moves into a negative electrical potential environment to bind into the pore of the channel in what is essentially a single step block-unblock reaction [6], [7]. Our results here further refine this hypothesis by providing evidence that the main N-type inactivation ball structure in Kv1 channels found throughout most of the animal kingdom (Kv1AnID) contains two highly conserved negative charges at positions 2 and 9. The presence of these negative charges clearly does not disrupt N-type inactivation rather they serve to shape the inactivation process in many important ways.

While substitution of uncharged residues into positions 2 and 9 can increase the apparent affinity of the ball peptide for the channel, the manner in which this occurs does not correspond to an increased efficiency for pore block. In all cases tested, uncharged substitutions into positions 2 and 9 resulted in excess binding affinity that did not correspond to increased pore block. Indeed, the native residue at position 2, glutamate, is found to be an amazingly optimal choice for N-type inactivation. Not only does E2 produce pore block more effectively than any other residue tested, it also does so in a manner that highly efficient. The position of E2 on the predicted curves in Figs. 9A–B suggests that for this residue all binding energy is being converted into pore block. While this is level of efficiency is essentially matched by E2D and E2K, both substitutions are relatively ineffective in producing pore block. Residues E2T and E2A show components of pore block that are highly efficient, but they also contain a second inefficient component. For E2A, 65% of the channels do recover in an efficiently blocked manner, possibly explaining why this residue is a common choice in insect Kv1 inactivation ball peptides, such as ShB [7]. For position 9, blocking efficiency is nearly optimal with either E9 or E9K suggesting that highly efficient pore block only requires a charge at this position rather than a single specific residue. This observation is supported by the evolutionary data showing that in addition to glutamate at position 9 some channels in this evolutionary family substitute in aspartate and lysine (Fig. 1). Our hypothesis then is that evolution is selecting for residues at positions 2 and 9 of the Kv1AnID that produce optimally effective and efficient pore block.

Our studies also show that residue 2 moves deeply into the channel pore in the inactivation Block state, experiencing 35% of the applied electric field. Although residue 2 is the only charged residue in the initial N-terminus, there appears to be a hidden positive charge that is experiencing a similar fraction of the electric field as residue 2 at both the ON Threshold step and the Bound state. The most likely explanation for this effect is that the N-terminus is unmodified in the Kv1AnID sequence and thus has a free, positively charged N-terminal amino group. Given that a large number of metazoan proteins are N-terminally modified, it seems possible that some of the sequence conservation in the Kv1AnID is to prevent interactions with enzymes that modify N-termini, rather than to block the pore, per se.

Given our hypothesis that residue 2 is negatively charged and near a positively charged N-terminus in the Bound state, it seems likely that significant electrostatic interactions might be occurring between these charges since the environment around this site is expected to be largely non-polar. If so, this could easily explain the 4.2 kT lower pore blocking affinity for E2D in the pore block site (K_I_ (0 mV): E2 = 51.3±0.3 (11); E2D = 0.79±0.3 (3)) since this shorter side chain would be expected to be at least 1 Å further from the N-terminal charge. Rough structural modeling of the N-terminus suggest that the charge on E2 could easily be within 4.5 Å of the N-terminal charge, and possibly much closer, with the charge on E2D being ∼1 Å further. If we use these numbers as a first estimate, the energy difference in water (ε_r_ = 80) would be only 0.8 kT for the E2D substitution. However in a more hydrophobic environment, such as the channel core (ε_r_ = 10) the energetic cost for E2D is 6.2 kT. This difference in energy is sufficient to explain the entire effect of substituting E2D into the channel. Importantly, this effect occurs without invoking any specific interactions between E2 and the channel core. For E2Q and E2N we can imagine similar, but weaker, hydrogen bond energy differences between these polar R-groups and the hypothesized N-terminal charge to explain the difference in apparent greater blocking affinity for E2Q at positive potential.

**Figure 11:**
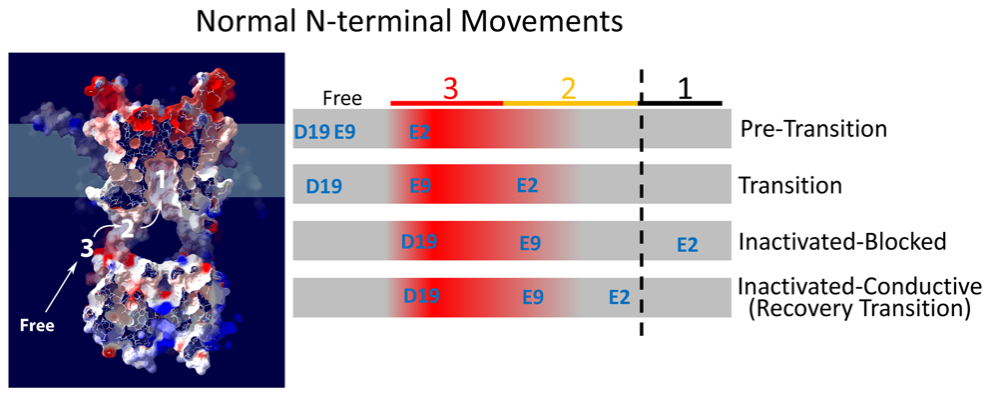
Model of Important Sites and Reaction Steps during N-type Inactivation. Structural model of the AKv1 channel showing the approximate locations of Sites 1, 2, and 3 along the internal aqueous pathway leading to the selectivity filter. Channel profile is taken from a slab cut from an AKv1 structural model showing a side window opening and the internal vestibule of the transmembrane pore. The 1.4 Å accessible surface map is colored according to electrostatic potential with red negative, white neutral, and blue positive. Schematic to the right lists the hypothesized locations of residues E2, E9 and D19 during different phases of the N-type inactivation cycle. The intensity of red indicates the approximate level of negative charge along the pathway relative to residues 161–3. Dashed vertical line indicates approximate location of the pore entry beyond which chain occupancy produces pore block. This picture provides an approximate explanation for the electrostatic coupling seen between these residues and 161–3 during the inactivation cycle. Precisely how this electrostatic field is actually experienced by any residue along the path will depend on the microenvironment of the N-terminal residue and the direction the residue is pointing relative to residues 161–3.

In fact, all the effects ascribed to residue 2 substitutions could be explained by a competition between two locations, one deep within the pore (Site 1) and one near the opening of the pore exposed to water (Site 2) (Fig. 11). Site 1 would be the pore blocking site, 35% of the way into the transmembrane electric field, which has a largely hydrophobic environment. Site 2 could be the ON Transition state site for residue 2, which experiences much less of the transmembrane electric field (15%), has a more negative electrostatic potential than Site 1 (−15.9 mV compared to −1.4 mV) and binds non-charged residues better than charged by about 1.5 kT. Weak blocking of the channel would be caused by a preference for the intermediate Site 2 compared to Site 1, also likely producing the vertical shift in the Linear Brønsted plot for mutations at residue 2 other than E2D (Fig. 5D). Voltage-dependence to block would be produced by the greater electric field felt at Site 1 compared to Site 2, shifting the relative equilibrium between these two sites. Non-polar substitution at position 2 would be expected to produce delayed recovery with multiple kinetics due to binding at Site 2. Indeed the very slow recovery for E2N and E2Q could be explained if channel closing partially traps the N-terminus at Site 2, in a manner that does not occur for other position 2 substitutions. Channel reopening would then appear to produce a non-inactivating current since the N-terminus is already bound to a site beyond the normal ON Transition state. For E2K, weak block is likely due to the energetic costs of moving 2 positive charges into Site 1, a non-polar pore location, compared to sitting at the water accessible, electrostatically negative Site 2. E2K likely recovers efficiently, however, since the side window (Site 3) potential is even more negative and a Site 2 binding interaction apparently does not occur with charged residues at position 2 (Fig. 11).

Position 9 residues appear to be located at or near Site 2 in the pore blocked state: a negative electrostatic environment that prefers interactions with non-charged residues by about 1.5 kT. However, mutations that enhance binding of residue 9 to Site 2 also reduce pore block efficiency. If the N-terminal structure between positions 2 and 9 were rigid in the Bound state, then the binding of position 9 with the channel core would be associated with highly efficient pore block, even though its interaction with the channel would technically be outside the actual pore block site. The decreased efficiency seen with the uncharged position 9 substitutions is strong evidence that the N-terminus retains sufficient flexibility between positions 2 and 9, to allow some independence of movement between these two residues and thus at most only partially convert the additional binding affinity of uncharged residue 9 substitutions into more efficient pore block. Sequence conservation shows that small, flexible residues are highly conserved between residues 2 and 9 in agreement with this hypothesis (Fig. 1).

It therefore seems likely that the properties we are ascribing to Site 2 are in fact a general description of much of the environment within the channel between the side window opening and the pore block site, rather than a single competitive site. In the Blocked state, residue 9 would then be located at a more peripheral position in the Site 2 region and residue 2 would be jumping between a more axial location in Site 2 and Site 1 to produce pore block (Fig. 11). An important unanswered question however is whether any of the other uncharged residues in the N-terminus normally bind to Site 2 in a manner that promotes efficient pore block. It is possible that negative charges inserted in other residues could disrupt other interactions with Site 2 that promote efficient pore block, potentially explaining the L7E mutation and the effects of phosphorylation.

The model we have converged upon is very similar to the Pre-Inactivation site model proposed by Zhou et al, 2001 [32]; however, we propose multiple potential interaction sites leading from the side window openings to the pore block site where the important of any specific site depends upon the types of residues present in the N-terminus [17]. The strong negative charge at Site 3, largely produced by EDE161-3, is critical for bringing the chain up to the side windows, by attracting chain positive charges R18, R26, R30, R38 and R42, and passing the N-terminal inactivation domain into the pore. E2 moving past the most negative region in Site 3 to Site 2 is the key ON Transition step (explaining the reversed coupling with charge at 161–3), while residue 9 is moving into Site 3, increasing its interaction with residues 161-3. Based on the similarities of the residue 9 environment in the Transition state to the residue 19 environment in the Bound state, it appears likely that as residue 2 moves into Site 1, residue 9 moves into Site 2 and residue 19 into site 3.

Generally speaking high levels of amino acid conservation are considered indicative of tight structural requirements for optimal interaction. In the case of the Kv1AnID, the conservation of charged residues at positions 2 and 9 is at least partly accounted for by their lack of interaction with the channel core site 2. This lack of interaction produces efficient block by ensuring that the interactions that do occur are causing pore block and not diverting the N-terminus from its optimal block site. We have furthermore hypothesized that E2 is highly conserved because it can move a negative charge closer to the amino terminal positive charge in the Bound State than E2D, lowering the energy for the N-terminus to occupy Site 1, rather than because it has optimal interactions with the non-polar residues lining the pore. Some studies have proposed a beta hairpin bend in the inactivation domain in the pore blocked state, with residue 1 at a relatively shallow location in the pore [12], [33], [34]. Our studies do not support such a model for the Kv1AnID. Residue 2 is 35% of the way across the electric field in the Bound state whereas residue 9 is 1%, meaning that a beta hairpin would likely have to be pushed entirely up through the selectivity filter to place residue 2 in such a deep location without also putting residue 9 in the pore. Given that the entire structure of the channel is designed to stabilize the selectivity filter within narrow limits, this structure seems entirely implausible. We therefore suggest that the extended N-terminal structural model proposed by Zhou et al. [32] is probably much more representative of the Kv1AnID structure in the Bound state than a beta hairpin.

## Supporting Information

Figure S1 **Conservation of Charged Residues in the Inactivation Domains of Kv channels and auxiliary subunits.** Negative charges are highlighted in green and positive in red. Conserved negative charges in positions 2 and 9 define the Kv1AnID as distinct from inactivation domains in Ecdysosa Kv1 channels. In Non-Kv1 alpha subunits and auxiliary subunits, other inactivation domain sequences are highly conserved within each family of related proteins, but do not share the same motif with the Kv1AnID. The high level of sequence conservation within each family suggests that inactivation domains are under strong evolutionary pressures once they have evolved.
(TIF)

Figure S2 **Three blocks of conserved sequence in Kv1 N-termini.** Sequences for Kv1 N-termini containing the Kv1AnID motif are aligned through the entire region prior to the T1 domain. In addition to the Kv1AnID motif, a characteristic secondary region of sequence conservation is evident (Block 2) along with additional sequence conservation leading into the T1 domain (Pre-T1). This alignment suggests that the entire N-terminus of this family is under significant evolutionary pressures, not just the inactivation domain.
(TIF)

Figure S3 **Two component tail current fittings of tail currents where residue 2 is uncharged.** Large trace shows the full command voltage and current response. Inset focuses on tail current. Black trace shows the recorded tail current. Red color shows the average amplitude of the tail from 0 current based on a filtered signal. Blue kinetic component is the two exponential fit to the tail current. The green line is the slower kinetic component which is predictive of the recovery kinetics for E2N, E2A and E2T. For E2A both tail current components are evident in the recovery from inactivation.
(TIF)
