# Supplementary figures and images for: Conserved N-Terminal Negative Charges Support Optimally Efficient N-type Inactivation of Kv1 Channels
Source: PLoS One. 2013 Apr 24;8(4):e62695. doi: 10.1371/journal.pone.0062695 (PMC3634772; doi:10.1371/journal.pone.0062695)

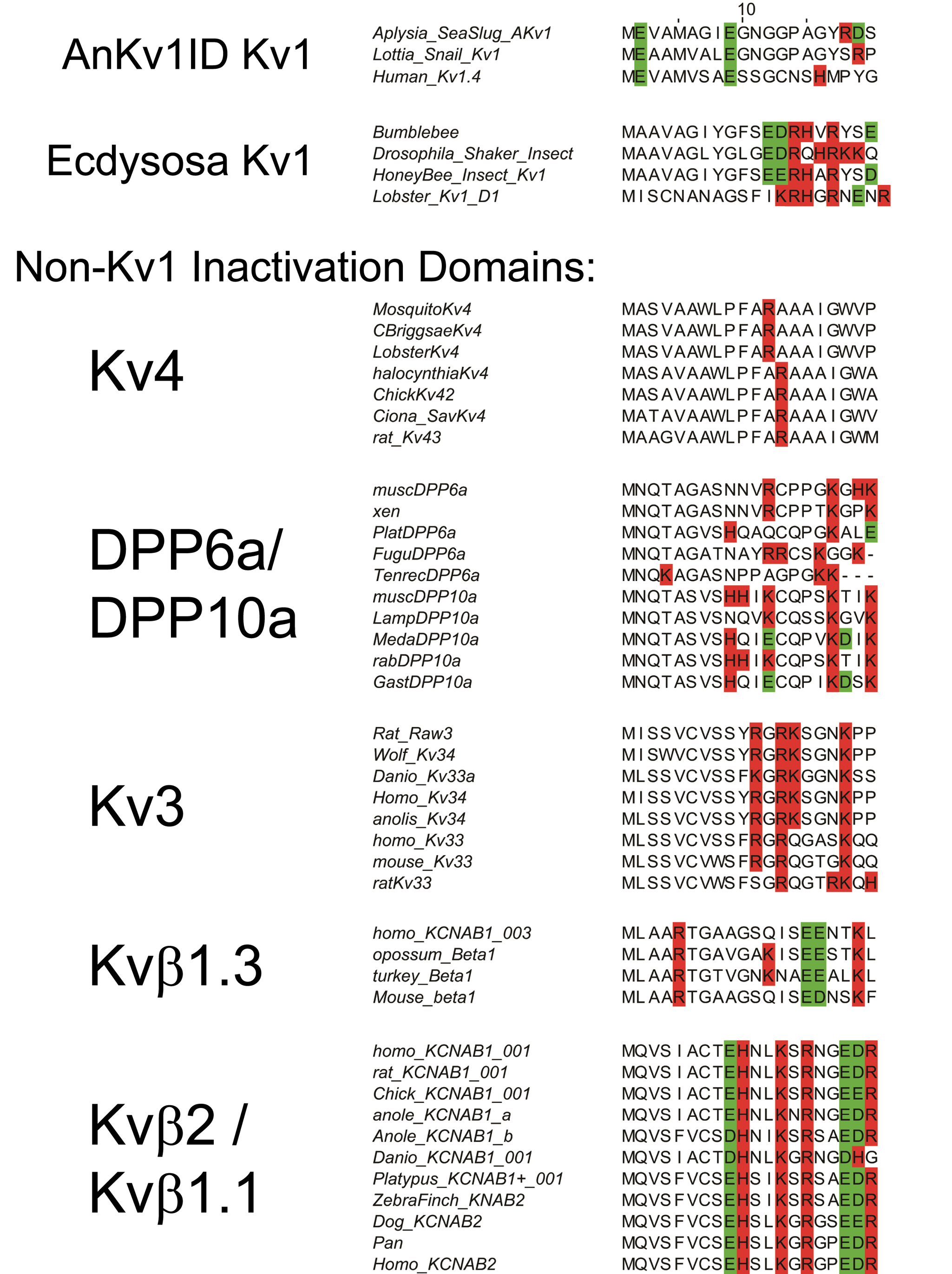

Supplement: Figure S1 — Conservation of Charged Residues in the Inactivation Domains of Kv channels and auxiliary subunits. Negative charges are highlighted in green and positive in red. Conserved negative charges in positions 2 and 9 define the Kv1AnID as distinct from inactivation domains in Ecdysosa Kv1 channels. In Non-Kv1 alpha subunits and auxiliary subunits, other inactivation domain sequences are highly conserved within each family of related proteins, but do not share the same motif with the Kv1AnID. The high level of sequence conservation within each family suggests that inactivation domains are under strong evolutionary pressures once they have evolved. (TIF) [file pone.0062695.s001.tif]

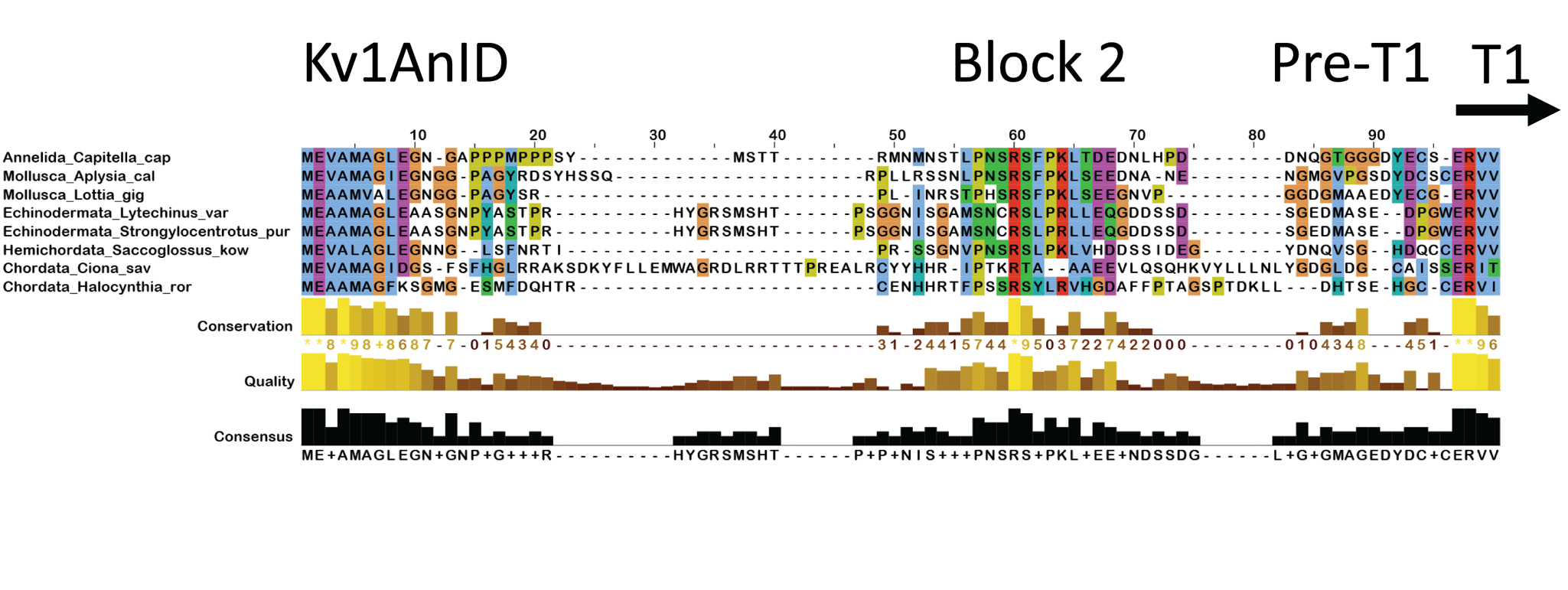

Supplement: Figure S2 — Three blocks of conserved sequence in Kv1 N-termini. Sequences for Kv1 N-termini containing the Kv1AnID motif are aligned through the entire region prior to the T1 domain. In addition to the Kv1AnID motif, a characteristic secondary region of sequence conservation is evident (Block 2) along with additional sequence conservation leading into the T1 domain (Pre-T1). This alignment suggests that the entire N-terminus of this family is under significant evolutionary pressures, not just the inactivation domain. (TIF) [file pone.0062695.s002.tif]

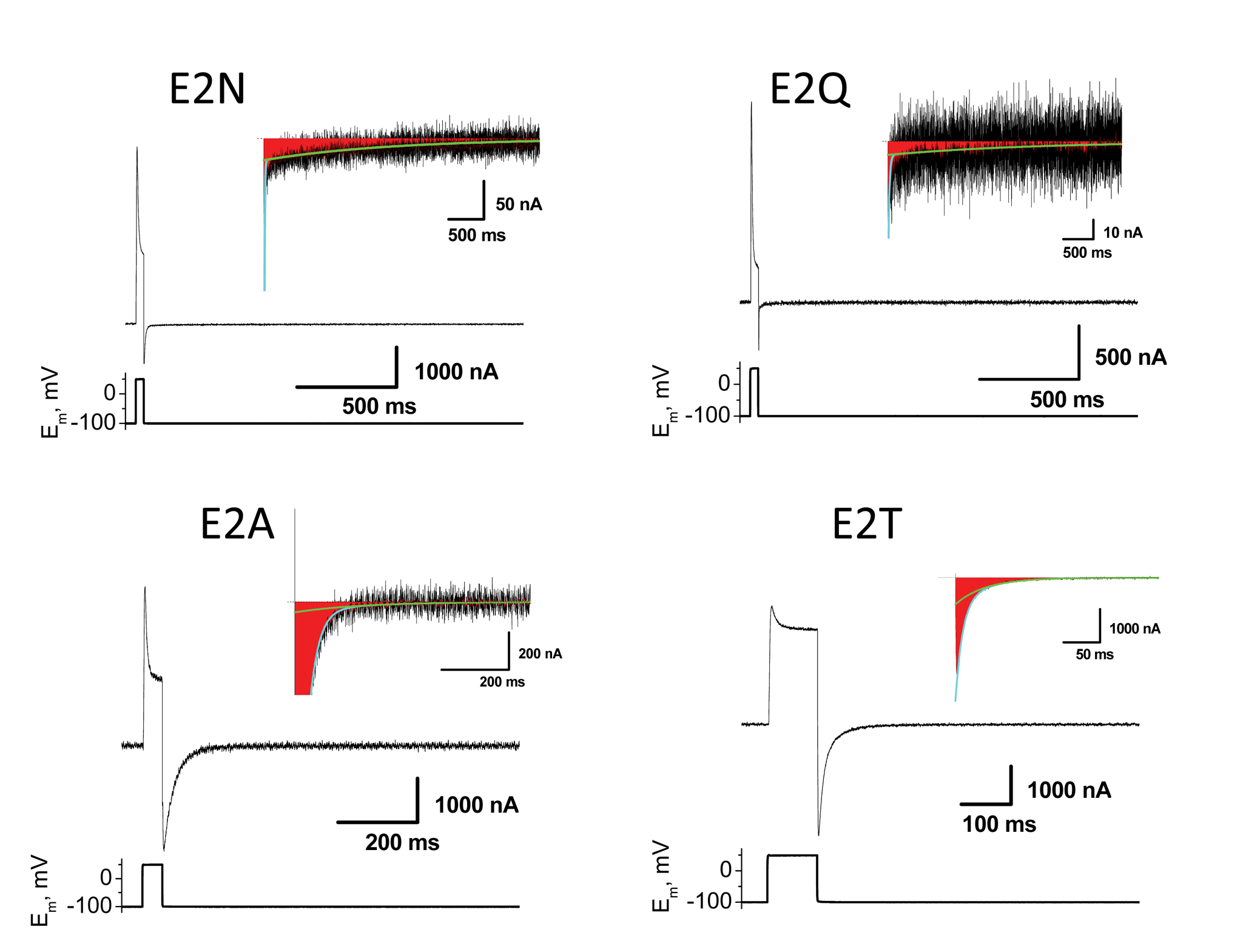

Supplement: Figure S3 — Two component tail current fittings of tail currents where residue 2 is uncharged. Large trace shows the full command voltage and current response. Inset focuses on tail current. Black trace shows the recorded tail current. Red color shows the average amplitude of the tail from 0 current based on a filtered signal. Blue kinetic component is the two exponential fit to the tail current. The green line is the slower kinetic component which is predictive of the recovery kinetics for E2N, E2A and E2T. For E2A both tail current components are evident in the recovery from inactivation. (TIF) [file pone.0062695.s003.tif]
